# Response of varied rice genotypes on cell membrane stability, defense system, physio-morphological traits and yield under transplanting and aerobic cultivation

**DOI:** 10.1038/s41598-023-32191-6

**Published:** 2023-04-08

**Authors:** Bassiouni A. Zayed, Hasnaa A. Ghazy, Mahrous E. Negm, Sherif M. Bassiouni, Adel A. Hadifa, Dalia E. El-Sharnobi, Mohamed M. Abdelhamed, Elsayed A. Abo-Marzoka, Amira M. Okasha, Salah Elsayed, Aitazaz A. Farooque, Zaher Mundher Yaseen

**Affiliations:** 1 Rice Research Department, Field Crops Research Institute, Agricultural Research Center, Kafr El-Sheikh, 33717 Egypt; 2 Crop Physiology Department, Field Crops Research Institute, Agricultural Research Center, Kafrelsheikh, 33717 Egypt; 3 Evaluation of Natural Resources Department, Environmental Studies and Research Institute, University of Sadat City, Minufiya, 32897 Egypt; 4 New era and Development in Civil Engineering Research Group, Scientific Research Center, Al-Ayen University, Thi-Qar, 64001 Baghdad, Iraq; 5 Canadian Center for Climate Change and Adaptation University of Prince Edward Island, St Peter’s Bay, PE Canada; 6 Faculty of Sustainable Design Engineering, University of Prince Edward Island, Charlottetown, PE Canada; 7 Civil and Environmental Engineering Department, King Fahd University of Petroleum & Minerals, Dhahran, 31261 Saudi Arabia; 8 Interdisciplinary Research Center for Membranes and Water Security, King Fahd University of Petroleum & Minerals, Dhahran, 31261 Saudi Arabia

**Keywords:** Plant biotechnology, Plant cell biology, Plant development, Plant signalling

## Abstract

Aerobic rice cultivation progresses water productivity, and it can save almost 50% of irrigation water compared to lowland rice with the appropriate development of genotypes and management practices. Two field trials were conducted during 2020, and 2021 seasons to determine the validation of different rice varieties under aerobic cultivation based on their plant defense system, physio-morphological traits, stress indices, grain yield, and water productivity. The experiments were designed in a split-plot design with four replications. Two planting methods, transplanting and aerobic cultivation, were denoted as the main plots, and ten rice genotypes were distributed in the subplots. The results revealed that the planting method varied significantly in all measured parameters. The transplanting method with well watering had the highest value of all measured parameters except leaf rolling, membrane stability index, antioxidant, proline, and the number of unfilled grains. EHR1, Giza179 and GZ9399 as well as A22 genotypes a chief more antioxidant defense system that operated under aerobic conditions. Giza179, EHR1, GZ9399, and Giza178 showed high cell membrane stability and subsequently high validation under such conditions, and also showed efficiency in decreasing water consumption and improving water use efficiency. In conclusion, this study proves that Giza179, EHR1, GZ9399, Giza178, and A22 are valid genotypes for aerobic conditions.

## Introduction

Rice (*Oryza sativa* L.) is an essential staple crop that secures food for almost two-thirds of the world's population^1^. The cultivation of rice occupies about twenty percent of the agricultural land area planted for cereals^2^. In Egypt, rice production faces many grand challenges such as freshwater scarcity, salt stress, the high cost of inputs, and overpopulation. It is estimated that rice's annual production deficit will increase from 400,000 t in 2016 to 800,000 by 2030^3^. Nowadays, the insufficient available freshwater for agriculture pose a challenge for big water consumer crops production (i.e., rice). The 79 million hectares of irrigated rice worldwide consume 34–43% of the irrigation water around the globe^4^.

Water resources management is one of the main factors affecting rice production globally^5^. Aerobic rice cultivation is a medium option to save water with an minimal yield reduction for irrigated rice ecotypes. Primarily, a water deficit may result in increasing spikelet sterility^6^. However, relative water content (RWC%) is easy to use for determining physiological status of plant under drought stress^7^. Also, RWC% is a vital guide to recognize the tolerant variety under various stresses such as aerobic cultivation with minimal water supply^8–13^ and salinity^13–18^. Decreasing freshwater resources are threatening agricultural production in many parts of the world^19^.

Plants suffer from various stresses, such as drought, low and high temperatures, salinity, etc.^20^. Among all, RWC%, leaf rolling, grain yield, leaf drying, root/shoot ratio, and root length offer high scope for rice development for aerobic cultivation^7^. There is an ever-increasing need to improve water productivity to stay commercially competitive. Abiotic stresses, namely, water deficit developed by aerobic cultivation or drought, are the most critical factors negatively influencing growth, and several physiological and biochemical processes^21^. Plants developed many adaptation mechanisms, such as antioxidant defense systems, i.e., Superoxide dismutase (SOD), Catalase (CAT), and Peroxidase (POD). These enzymatic components play a central role in the defense system. The SOD decomposes superoxide (O_2_) to H_2_O_2_ by catalyzing superoxide anion radical disputation. The POD and CAT decrease H_2_O_2_ in water using various substrates as electron donors to preserve the roots and help the plants endure stress^22–25^. Aerobic rice improves water productivity with the appropriate development of genotypes and management practices^7^. Aerobic rice is specially developed rice, combines drought tolerance of upland rice and yield potential in lowland rice^6^. Therefore, it can save almost 50% of irrigation water compared to lowland rice. Aerobic rice cultivars with high yield potential and moderate drought stress tolerance have been developed through traditional upland farming with improved irrigated cultivars^6^. Aerobic rice cultivars achieve high yields under appropriate management practices and deep root systems, as well as water stress tolerance in the vegetative and reproductive stages^26^.

The objectives of the current study were (1) to determine the physiological, morphological, and stress indices of some Egyptian rice varieties under aerobic cultivation; (2) the possibility to explore such type of cultivation in Egypt, and (3) to nominate valid Egyptian rice varieties to lead cultivation under aerobic conditions.

## Materials and methods

### Experimental site

The experiment was conducted at the experimental farm of Sakha Agricultural Research Station, Kafr El-Sheikh, Egypt, during 2020 and 2021 seasons to compare the performance of some rice varieties under normal transplanting and aerobic cultivation conditions. All experiments were preceded by a barley crop (*Hordeum* spp.). The results of the chemical soil properties are presented in Table 1.

**Table 1:** Mechanical and chemical analysis of the experimental soil during the two seasons.

Soil analysis	2020	2021
Soil texture	Clayey	clayey
pH	8.05	8.20
EC (dS m^−1^)	2.00	2.05
Organic matter %	1.65	1.50
Available NH_4_ (mg kg^−1^)	14.50	15.60
Available NO_3_ (mg kg^−1^)	12.00	13.80
Available P (mg kg^−1^)	15.00	12.00
Available K (mg kg^−1^)	280	270
Available Zn (mg kg^−1^)	1.15	1.16

The experiment was performed in a split plot design with four replications. The main plots were devoted to two planting methods: normal transplanting and aerobic cultivation, and subplots were occupied by ten varieties. As for the aerobic cultivation pattern, the dry seeds were used in dry land on furrows. The furrow size was 70 cm from mid-bottom of each of the two furrows, with a depth of 30 cm. Rice irrigation during the first 25 days of rice growth was done as in drill seeded rice, and then watering was done each eight days to maturity by filling the bottom of furrow. The recommended dose of nitrogen fertilizer (i.e., 165 kg N ha^−1^) was applied in three equal doses (basal, top dressing at panicle initiation, and late booting stage). For the permanent field, phosphorus fertilizer at the rate of 35.5 kg P_2_O_5_ ha^−1^ was basally applied to the soil during the land preparation. The potassium fertilizer (57 kg K_2_O ha^−1^) was added as a basal dose and incorporated into dry soil, and zinc (Zn SO_4_) was applied at the rate of 23.9 kg ha^−1^ before continuous flood irrigation. For transplanting cultivation method, the seeds of different rice varieties were sown on May, 7th, and after 30 days, the seedlings were transplanted in the permanent field at 20 × 20 cm plant spacing in a plot area of 10 m^2^. As for aerobic cultivation, the 4 dry seeds/hill were sown at the space of 15 cm between each pair of hills and 30 cm between the two rows on the both sides of furrow. Origin, parentage, and variety group are presented in Table 2.

**Table 2:** Origin, parentage, and variety group of the studied cultivars.

Rice genotype	Parentage	Type
1-Giza1779 (Chek sensetive)	Giza 171/Yomjo No.1//Pi No.4	Japonica
2-Sakha102	GZ 4096-7-1/Giza177	Japonica
3-Giza178 (chek tolerance)	Giza 175/Milyang 49	Indica/japonica
4-Giza179	GZ1368-S-5-4/GZ6296-12-1-2	Indica/Japonica
5-Sakha106	Giza177/Hexi30	Japonica
6-Sakha107	Giza177/BL1	Japonica
7-A22	IR47664	Indica
8-Egyptian Yasmine	IR262-43-8-11/KDML105	Indica
9-Egyptian hybrid1(EHR1)	IR69625A/Giza178	indica
10-GZ9399-4-1-1-3-2-2	Giza178/IR65844-29-1-3-1-2	Indica/Japonica

At heading stage, plants of five hills were randomly taken and pulled with their roots from each plot and transported to the lab to determine: leaf area index, flag leaf area using portable area meter membrane (Model LI-3000A), chlorophyll content (with SPAD meter M502), stability index, RWC%, length, and root volume. Also, some enzymatic antioxidants such as catalase, peroxidase, and super oxidase dismutase, and proline activity leaf content were assessed. In the field, leaf rolling was visually estimated according to the IRRI scale. The stomatal conductance was measured at head in the field. Root length and root volume was estimated as described by Bradford^27^. At harvest, plant height was estimated. The number of panicles on ten random hills was counted and then conformed to the number of panicles/hill. Ten random panicles were collected from each plot to estimate panicle length, number of filled grains/panicle, number of unfilled grains/panicles, panicle weight, 1000-grain weight and grain yield were randomly measured from an area of 1 m^2^ and adjusted to 14% moisture content.

### Determination of enzymatic activities

Leaf samples (i.e., 200 mg) were soaked in liquid N_2_ and homogenized in 2.0 ml of extraction buffer: 100 mm potassium phosphate (pH 7.8), 0.1 mm ethylene diaminetetra acetic acid (EDTA) and 10 mm ascorbic acid. The homogenate was centrifuged at 13,000*g* for 15 min at 4 °C. CAT activity was assayed in the supernatant at 240 nm based on the consumption of H_2_O_2_^28^. The activity of SOD was determined at 560 nm according to the method of Beauchamp and Fridovich^29^. Activity of POX was measured at 420 nm as described by Kar and Mishra^30^.

### Proline content

Leaf sample (0.3 g) were placed in 3% sulphosalicylic acid and centrifuged for 20 min at 3000×*g*. From extract, 2 mL of supernatant was added to 2 mL of ninhydrin reagent and 2 mL of glacial acetic acid. Proline was determined as mg g^−1^ FW using a spectrophotometer.

### Relative water content (RWC)

RWC was calculated according to González and González^31^ as the following:

1 $${\text{RWC}} = \frac{{[{\text{f}}.{\text{wt}} - {\text{d}}.{\text{wt}}]}}{{[{\text{t}}.{\text{wt}} - {\text{d}}.{\text{wt}}]}} \times 100$$

where f. wt. is the fresh weight of leaves, d. wt is the dry weight of leaves, t. wt is the turgid weight of leaves.

### Membrane stability index (MSI)

Youngest leaf tissues (0.2 g) were washed by deionized water, cut into sections (1 cm length), placed in 10 mL deionized water, and heated at 40 °C in a water bath for 30 min. Then the electrical conductivity (EC1) was measured by using a conductivity meter (ME977-C, Max Electronics). Subsequently, the content was boiled for 10 min in a boiling water bath (100 °C), and the conductivity (EC2) was measured. Finally, by using the formula described by Premachandra et al.^32^, the membrane stability index (MSI) was calculated as following equation

2 $${\text{MSI}} = \left[ {1 - \left( {\frac{{{\text{EC}}1}}{{{\text{EC}}2}}} \right)} \right] \times 100$$

### Stomatal conductance (GS)

The stomatal conductance (gs) (units; mol m^−2^ s^−1^) was measured at the heading stage via a portable photosynthesis measurement system (Li-Cor, Lincoln, NE, USA) according to Hubbard et al.^33^.

### Statistical analysis

Data collected were statistically analyzed using the analysis of variance technique according to Gomez and Gomez^34^. Duncan's Multiple Range Test was used to compare the treatment means^35^. All statistical analyses were accomplished using the analysis of variance technique using the "COSTAT" statistical software package^36^.

### Experimental research

The current experiment including all studied materials tightly comply with relevant plant guidelines and legislation at various levels.

## Results

### Antioxidant enzymes activity

The primary indicator of drought or low water supply for rice crop was releasing freer radical which could be treated by antioxidants formation. Notably, the activity of catalase, peroxidase, superoxide dismutase, and proline were significantly influenced by planting methods in both seasons (Table 3).

**Table 3:** Some antioxidants and proline of some rice genotypes affected by the planting methods.

Treatment	Catalase (µmol min^−1^ g^−1^ protein)		Peroxidase (µmol min^−1^ g^−1^ protein)		SOD (µmol min^−1^ g^−1^ protein)		Proline (mgg^−1^ FW)	
	2020	2021	2020	2021	2020	2021	2020	2021
Planting method (T)								
Transplanting rice	0.069b	0.070b	1.44b	1.36b	0.269b	0.260b	2.17b	2.23b
Aerobic cultivation	0.107a	0.118a	1.94a	1.89a	0.386a	0.371a	19.89a	20.15a
F. test	*	*	**	**	**	**	**	**
Rice genotype (V)								
1-Giza177	0.049gh	0.056d	1.17f.	1.21f.	0.205 g	0.201f.	6.76f.	6.48f.
2-Sakha102	0.052 g	0.051d	1.24ef	1.30ef	0.218 g	0.205f.	8.10e	8.34e
3-Giza178	0.111c	0.116b	1.50c	1.75c	0.360d	0.355c	13.47b	14.07b
4-Giza179	0.123b	0.118b	2.06b	2.09b	0.443c	0.433b	13.92b	14.35ab
5-Sakha106	0.046 h	0.045d	1.23ef	1.22f.	0.155 h	0.128 g	6.60f.	6.80f.
6-Sakha107	0.068f.	0.070 cd	1.31e	1.30ef	0.281f.	0.281e	10.74d	11.26c
7-A22	0.103d	0.103bc	1.27e	1.32e	0.305e	0.315d	11.77c	11.39c
8-E. Yasmine	0.072e	0.073 cd	1.50d	1.47d	0.295ef	0.286e	10.50d	9.90d
9-EHR1	0.128a	0.138ab	2.57a	2.48a	0.526a	0.493a	14.76a	15.10a
10-GZ9399	0.129a	0.166a	2.52a	2.23a	0.486b	0.450b	13.65b	14.13b
F. test	**	**	**	**	**	**	**	**
Interaction (V × T)	**	NS	**	**	**	**	**	**

Means followed by a common letter at the same column are not significantly different according to Duncan^’^s multiple range at 0.05 levels.

*Significant at 0.05 level.

**Significant at 0.01 level and NS = Not significant.

Aerobic cultivation conditions shows the highest values of catalase, peroxidase, superoxide dismutase, and proline compared with the transplanting method in both seasons. The tested rice varieties show remarkable variation in their antioxidant defense systems. EHR1 and GZ9399 rice genotypes were at the same level and recorded the highest value of catalase and peroxides, followed by Giza179 and Giza178 in both seasons. As for SOD and proline accumulation, EHR1 values were the highest, followed by GZ9399, Giza179 and Giza178. The interaction between planting methods and varieties effect significantly on catalase, peroxidase, proline, and superoxide dismutase (Figs. S1–S8). EHR1 surpassed in production of antioxidant system under aerobic cultivation method followed by GZ9399 and Giza179. Giza178, A22, Egyptian Yasmine and Sakha107 showed medium values of antioxidants and proline accumulation under aerobic cultivation (Figs. S1–S8). However, the lowest values of studied antioxidants and proline accumulation under aerobic cultivation were produced by Giza177, Sakha102 and Sakha106 varieties in both seasons.

### Physio-morphological traits

Leaf area index, flag leaf area, and leaf rolling were significantly influenced by the cultivation methods in both seasons (Table 4). The two planting methods showed an apparent variation in both seasons. The transplanting method with well-watering treatment possessed the highest values of the leaf area index and flag leaf area, and the lowest values of leaf rolling. Therefore, aerobic cultivation showed a slight reduction in vegetative and physiological growth traits.

**Table 4:** Growth parameters of some rice genotypes affected by the planting methods.

Treatment	Leaf area index		Flag leaf area (cm^2^)		Leaf rolling	
	2020	2021	2020	2021	2020	2021
Planting method (T)						
Transplanting rice	6.56a	6.58a	30.36a	30.51a	2.60b	2.73b
Aerobic cultivation	4.54b	4.56b	21.20b	21.84b	4.57a	4.70a
F. test	**	**	**	**	**	**
Rice genotype (V)						
1-Giza177	4.68 g	4.66e	21.31 fg	20.82 g	5.16a	5.07a
2-Sakha102	4.70 g	4.90f.	23.13ef	23.46f.	5.00a	5.09a
3-Giza178	5.63e	5.83d	25.06cde	26.55d	2.66c	3.05 cd
4-Giza179	5.80de	5.75d	29.95b	29.46b	2.00c	2.50de
5-Sakha106	4.55 g	4.43 g	20.65 g	21.18 g	4.83a	5.16a
6-Sakha107	5.10f.	5.08e	25.83 cd	25.48e	3.50b	3.83b
7-A22	5.88 cd	5.76d	24.05de	25.10e	3.66b	3.33bc
8-E. Yasmine	6.05c	6.11c	26.70c	28.01c	4.00b	3.94b
9-EHR1	6.70a	6.80a	35.11a	34.98a	2.50c	2.16e
10-GZ9399	6.38b	6.20b	25.98 cd	26.63	2.67c	2.99 cd
F test	**	**	**	**	**	**
Interction (V × T)	**	**	**	**	**	**

Means followed by a common letter at the same column are not significantly different according to Duncan^’^s multiple range at 0.05 levels.

*Significant at 0.05 level.

**Significant at 0.01 level.

The tested genotypes significantly varied in their leaf area index, flag leaf area, and leaf rolling in both seasons. EHR1 had the largest leaf area index and flag leaf area, followed by GZ9399 in leaf area and Giza179 in flag leaf area. Giza178 and Egyptian Yasmine occupied the second rank regarding the above-mentioned traits. The known sensitive rice varieties to drought, such as Giza177, Sakha102, and Sakha106, showed the narrowest leaf area index and flag leaf area in both seasons. Leaf rolling peaked with Giza177 and Sakha106 rice varieties in the first and second seasons, respectively. In both seasons, EHR1 had the lowest means of leaf rolling in the two seasons, respectively. Results in Table 5 show that the interaction effect between planting methods and rice genotypes on leaf area index and flag leaf area was significant in both seasons.

**Table 5:** Leaf area index and flag leaf area affected by the interaction between the study factors.

Treatment	Leaf area index				Flag leaf area (cm^2^)			
	2020		2021		2020		2021	
Planting method (T)								
Rice genotype (V)	T1	T2	T1	T2	T1	T2	T1	T2
1-Giza177	6.16de	3.16i	6.34de	3. 23jk	28.20de	14.43 g	27.45 g	14.20 h
2-Sakha102	6.23d	3.20i	6.16ef	3.46j	31.66 cd	15.10 g	30.76de	16.16 m
3-Giza178	6.63bc	4.63 g	6.76bc	4.90gh	28.89de	21.23f.	30.10ef	23.00j
4-Giza179	6.53c	5.06f.	6.56 cd	4.93gh	32.33bc	27.56de	32.16c	26.76gh
5-Sakha106	6.00de	3.10i	5.86f.	3.00 k	26.30e	15.00 g	25.86hi	16.50 m
6-Sakha107	6.10de	4.10 h	5.96f.	4.20i	31.66 cd	20.00f.	31.76 cd	19.20 l
7-A22	6.66bc	5.10f.	6.76bc	4.76 h	27.95de	20.16f.	29.50f.	20.70 k
8-E. Yasmine	6.76bc	5.33f.	7.06b	5.16 g	33.06b	20.33f.	33.86b	22.16j
9-EHR1	7.50a	5.90de	7.50a	6.10ef	36.66a	33.56b	35.63a	34.33b
10-GZ9399	7.00b	5.76e	6.83bc	5.86f.	27.33de	24.63e	27.93 g	25.33i
F test	**		**		**		**	

T1 = Transplanting, T2 = Aerobic, **Significant at 0.01 level. Means followed by a common letter at the same column are not significantly different according to Duncan^’^s multiple range at 0.05 levels.

EHR1 gave the highest leaf area index and flag leaf values when it was transplanted with well-watering compared with the other treatments. Whereas, the lowest leaf area index values were produced by Sakha106 under the aerobic conditions without significant differences with Giza177 and Sakha102 under the same conditions. The interaction effect showed the ability of EHR1, GZ9399, Giza179 and Giza178 to grow well under aerobic conditions as compared to the rest of the rice genotypes considering leaf area index and flag leaf area. The interaction effect between planting methods and varieties on leaf rolling was significant in both seasons. The tested genotypes exhibited great and marked differences, particularly under aerobic cultivation. Under the transplanting method, the tested genotypes were at par regarding leaf rolling in both seasons of the study. EHR1 and Giza179 showed the lowest leaf rolling under aerobic cultivation in both seasons, followed by GZ9399 and then Giza178. Egyptian Yasmine, A22, and Sakha107 had medium leaf rolling when cultivated under aerobic conditions. Giza177, Sakha106, and Sakha102 exhibited the highest leaf rolling under aerobic conditions, supporting their sensitivity to water deficit in both seasons (Table 6).

**Table 6:** Leaf rolling affected by the interaction between the study factors.

Treatment	Leaf rolling			
	2020		2021	
Planting method (T)				
Rice genotype (V)	Transplanting (T1)	Aerobic (T2)	Transplanting (T1)	Aerobic (T2)
1-Giza177	3.00c	7.33a	3.33cd	6.82a
2-Sakha102	3.00c	7.00a	3.00cde	7.18a
3-Giza178	2.33c	3.00c	2.66de	3.44cd
4-Giza179	2.00c	2.00c	2.33de	2.66de
5-Sakha106	2.66c	7.00a	3.00cde	7.33a
6-Sakha107	2.33c	4.66b	2.66de	5.00b
7-A22	3.00c	4.33b	2.66de	4.00bc
8-E. Yasmine	2.66c	5.33b	3.00cde	4.89b
9-EHR1	2.33c	2.66c	2.000e	2.33de
10-GZ9399	2.66c	2.68c	2.66de	3.33cd
F test		**		**

**Significant at 0.01 level. Means followed by a common letter at the same column are not significantly different according to Duncan^’^s multiple range at 0.05 levels.

As seen in Table 7, aerobic cultivation with less irrigation waterhave increased water loss through stomata and high transpiration rate, resulting in low leaf water content, low chlorophyll content, and the highest electrolyte leakage (EL%) in terms of cell membrane stability index values. On the other hand, the transplanting method with well watering had the highest mean of RWC and chlorophyll content and the lowest values of the membrane stability index in both seasons. GZ9399 gave the lowest values of EL% in the terms of cell membrane stability index without significant differences with Giza179 in both seasons and EHR1 in the second season. Sakha106 had a higher in cell membrane stability index and Giza177 in the second season, followed by Sakha102. However, Sakha107, A22, and Egyptian Jasmine showed a medium rate of cell membrane stability. It is mentioned here that the high stability index meant a higher EL%, indicating the sensitivity of variety.

**Table 7:** Membrane stability index, leaf relative water content and chlorophyll of rice genotypes affected by planting methods**.**

Treatment	Membrane stability index (%)		RWC (%)		Chlorophyll content	
	2020	2021	2020	2021	2020	2021
Planting method (T)						
Transplanting rice	3.20b	3.21b	89.33a	88.69a	37.10a	38.20a
Aerobic cultivation	12.19a	13.27a	77.24b	75.40b	30.05b	30.03b
F. test	**	**	**	**	**	**
Rice genotype (V)						
1-Giza177	12.03b	13.10a	71.58g	69.93g	31.60ef	32.15d
2-Sakha102	11.41b	11.66b	80.13f	77.78f	32.41e	32.30d
3-Giza178	5.60d	6.10ef	86.33b	85.91b	33.50d	35.43b
4-Giza179	4.76e	5.23fg	89.48a	88.41a	35.27c	36.58b
5-Sakha106	12.91a	13.76a	81.86e	78.06ef	29.01g	30.88d
6-Sakha107	7.16c	8.23c	82.14de	80.06de	34.51cd	33.99c
7-A22	6.34cd	6.65de	83.87cd	83.28c	30.76f	32.11d
8-E. Yasmine	6.85c	7.56cd	83.34de	81.83cd	31.33ef	31.66d
9-EHR1	5.60d	5.48fg	84.76bc	86.22b	40.06a	39.28a
10-GZ9399	4.15e	4.58g	89.36da	88.40a	37.27b	36.73b
F. test	**	**	**	**	**	**
Interaction (V × T)	**	**	**	**	**	**

**Significant at 0.01 level. Means followed by a common letter at the same column are not significantly different according to Duncan^’^s multiple range at 0.05 levels.

The low index of EL% is favorable because of low cell membrane damage. Giza179 rice variety had the maximum values of RWC%, followed by GZ9399 without significant differences in both study seasons. Giza177 rice variety gave the lowest values of RWC in the 2020 and 2021 seasons, followed by Sakha102 and Sakha106. The EHR1 came in advanced rank considering leaf RWC. Interestingly, Leaf chlorophyll content was high in EHR1 followed by GZ9399 and then Giza179 and Giza178. Whereas, the minimum averages of chlorophyll content were observed in Sakha106 followed by Giza177 and Sakha102 in both seasons (Table 5). The interaction effects between planting methods and rice genotypes on membrane stability index, RWC, and chlorophyll content were significant in both seasons of the study (Figs. S7, S8 and Table 6). The tested genotypes were apparently varied in both seasons under the transplanting method regarding to RWC and cell membrane stability index. Under aerobic cultivation, Sakha106 rice variety gave the highest values of membrane stability index followed, by Giza177 and Sakha102 under aerobic conditions indicating their unsuitability for that cultivation method. The previously mentioned varieties showed the same behavior with leaf RWC%. Giza177 gave the lowest values of RWC% and chlorophyll content followed by Sakha106, then Sakha107 under aerobic cultivation. GZ9399 gave the highest values of RWC% under aerobic, followed by Giza179 without significant differences in both seasons that was completely matching with their cell membrane stability (Figs. S7 and S8), EHR1 under both aerobic and transplanting methods gave the highest values of chlorophyll content in both seasons (Table 8).

**Table 8:** Leaf relative water content and chlorophyll content affected by the interactions.

Treatment	Leaf relative water content (%)				Chlorophyll content			
	2020		2021		2020		2021	
Rice genotype	T1	T2	T1	T2	T1	T2	T1	T2
1-Giza177	81.70d	61.47g	80.29d	59.56i	39.21c	24.00j	40.03ab	24.26k
2-Sakha102	83.70d	76.56ef	82.20cd	73.37gh	39.70bc	25.13j	40.14ab	24.46k
3-Giza178	89.37bc	83.28d	90.43a	81.39cd	35.00ef	32.00g	37.52cd	33.33g
4-Giza179	91.49ab	87.47c	92.00a	84.83bc	36.55de	34.00fg	37.96bc	35.20efg
5-Sakha106	88.69bc	75.03ef	86.25b	69.88h	32.93g	25.10j	35.36ef	26.40j
6-Sakha107	88.63bc	75.66ef	86.81b	73.30gh	41.13ab	27.90i	40.85ab	27.13ij
7-A22	90.20bc	77.54e	90.70a	75.86ef	33.20fg	28.33i	35.33ef	28.70hi
8-E. Yasmine	93.38a	73.30f	92.26a	71.40gh	32.60g	30.06h	34.03fg	29.29h
9-EHR1	94.39a	75.13ef	94.06a	78.37de	42.13a	38.00cd	41.40a	37.16de
10-GZ9399	91.68ab	87.03c	90.98a	86.00b	38.55c	36.00e	39.14bc	34.33fg
F. test	**			**		**		**

T1 = Transplanting, T2 = Aerobic, **Significant at 0.01 level. Means followed by a common letter at the same column are not significantly different according to Duncan^’^s multiple range at 0.05 levels.

Results in revealed that stomata conductance, root length, and root volume were significantly influenced by planting methods in both seasons (Table 9). The transplanting method with well-watering treatment possessed the highest stomata conductance, root length, and root volume values. In the tested rice genotypes, there were significant differences in stomata conductance, root length, and root volume in both seasons. Further, rice varieties significantly differed in their root length and root volume.

**Table 9:** Stomata conductance, root length and root volume of some rice genotypes affected by the planting methods.

Treatment	Stomata conductance (mmol m^−2^ s^−1^)		Root length (cm)			Root volume (cm^3^)	
	2020	2021	2020	2021		2020	2021
Planting method (T)							
Transplanting rice	977.17a	977.33a	29.89a		30.07a	65.60a	66.55a
Aerobic cultivation	740.50b	733.07b	22.51b		23.14b	48.92b	50.69b
F. test	**	**	**		**	**	**
Rice genotype (V)							
1-Giza177	761.16g	766.16g	19.68g		20.58h	40.07h	41.96j
2-Sakha102	768.00g	772.33g	21.58f		21.87g	49.42g	49.03i
3-Giza178	893.83bc	895.83b	29.16c		30.11bc	59.30d	61.50e
4-Giza179	912.50a	905.83a	29.62bc		30.67b	66.58b	66.62c
5-Sakha106	858.50e	845.50e	24.68e		24.85f	55.68e	56.53f
6-Sakha107	872.50d	862.33d	25.89d		26.05e	52.02f	53.82g
7-A22	885.00c	875.66c	26.49d		27.45d	61.34c	63.56d
8-E. Yasmine	820.83f	825.83f	22.00f		22.31g	48.81g	50.36h
9-EHR1	913.33a	907.50a	32.80a		32.51a	71.53a	73.56a
10-GZ9399	902.66ab	895.00b	30.05b		29.65c	67.76b	69.23b
F. test	**	**	**		**	**	**
Interaction (V × T)	**	**	**		**	**	**

**Significant at 0.01 level. Means followed by a common letter at the same column are not significantly different according to Duncan^’^s multiple range at 0.05 levels.

As for the genotypes, EHR1 had much more profound and extensive root distributions than Giza177 in both seasons. The maximum values of stomata conductance was shown by EHR1 and Giza179 rice varieties without any significant differences among them, followed by GZ9399 in the two seasons. However, the known sensitive rice varieties, such as Giza177, Sakha102 showed the lowest values of stomata conductance in both seasons of study. Results in Table 10 shows that the interaction effect between planting methods and varieties on root length, root volume, and stomata conductance was significant in both study seasons. EHR1 gave the highest values of root length and volume under aerobic rice cultivation followed by Giza179 (Table 10).

**Table 10:** Root length, root volume and stomata conductance as affected by the interaction between rice genotypes and planting methods.

Treatment	Root length (cm)				Root volume (cm^3^)			
	2020		2021		2020		2021	
Planting method (T)								
Rice genotype (V)	T1	T2	T1	T2	T1	T2	T1	T2
1-Giza177	25.31g	14.05l	26.13gh	15.03h	54.24hi	25.91m	55.70g	28.22k
2-Sakha102	27.79ef	15.38k	27.35fg	16.40m	56.76gh	42.08l	57.04fg	41.03j
3-Giza178	31.99c	26.33g	32.63b	27.60fg	69.01d	49.59j	70.74c	52.26h
4-Giza179	31.84c	27.40ef	32.85b	28.50ef	72.20c	60.97ef	71.96c	61.27e
5-Sakha106	28.76de	20.60i	27.59fg	22.10k	61.27ef	50.10j	60.40e	52.66h
6-Sakha107	28.80de	22.97h	29.66de	22.43jk	59.23fg	44.80k	60.98e	46.66i
7-A22	29.54d	23.44h	31.26c	23.63ij	69.47d	53.20i	71.43c	55.70g
8-E. Yasmine	25.24g	18.77j	24.83hi	19.80l	58.07fg	39.55l	58.12f	42.60j
9-Hybrid1	36.07a	29.53d	34.50a	30.53cd	79.29a	63.78e	81.26a	65.86d
10-GZ9399	33.50b	26.60fg	33.90ab	25.40h	76.38b	59.15fg	77.86b	60.60e
F test	**	**			**		**	

Treatment	Stomata conductance (mmol m^−2^ s^−1^)							
	2020		2021					
Planting method (T)								
Rice genotype (V)	Transplanting	Aerobic	Transplanting	Aerobic				
1-Giza177	964.00cd	558.33k	970.00bc	362.33j				
2-Sakha102	974.33abc	561.66k	979.66ab	365.00j				
3-Giza178	982.66ab	805.00g	985.00a	806.66e				
4-Giza179	985.00ab	840.00e	986.66a	825.00d				
5-Sakha106	978.66abc	738.33i	974.33abc	716.66h				
6-Sakha107	981.66ab	763.33h	978.00ab	746.66g				
7-A22	973.33bc	796.66g	968.00bc	783.33f				
8-E. Yasmine	956.66d	685.00j	961.66c	690.00i				
9-EHR1	990.00a	836.66e	986.66a	828.33d				
10-GZ9399	985.33ab	820.00f	983.33a	806.66e				
F. test		**		**				

T1 = Transplanting, T2 = Aerobic, **Significant at 0.01 level. Means followed by a common letter at the same column are not significantly different according to Duncan^’^s multiple range at 0.05 levels.

The lowest mean value of the root length and root volume was produced by Giza177 under the aerobic conditions followed, by Sakha102 and Egyptian Yasmine in both seasons. Under the transplanting method, the majority of rice genotypes did not show remarkable differences regarding the studied root characteristics (Table 8). The interaction between rice genotypes and planting methods is significantly affected stomata conductance in both seasons.

Under the transplanting method, EHR1 gave the maximum values of stomata conductance without significant differences from those obtained by GZ9399, Giza179, Giza178, Sakha107, Sakha102 and Sakha106 in, while the lowest values were recorded by E. Yasmine followed by Giza177 and A22 in both season (Table 8). Under aerobic cultivation, Giza179 exerted the highest mean of stomata conductance, followed by EHR1 with the same level of significance.

### Yield and yield components

The planting methods significantly influenced plant height, number of panicles/hills, and panicle length in both seasons as shown in Table 11. It was clear that the highest values of traits were recorded by the transplanting method compared to aerobic cultivation in both seasons. It was observed that rice genotypes significantly varied in their plant height, number of panicles/hills, and panicle length in both seasons.

**Table 11:** Some yield attributes of some rice genotypes affected by the planting methods during the two seasons.

Treatment	Plant height (cm)		Number of panicle/hill		Panicle length (cm)	
	2020	2021	2020	2021	2020	2021
Planting method (T)						
Transplanting rice	97.31a	97.27a	28.02a	27.33a	21.76a	21.95a
Aerobic cultivation	85.15b	85.77b	18.05b	18.70b	18.85b	19.01b
F. test	**	**	**	**	**	**
Rice genotype (V)						
1-Giza177	85.50d	85.83f	18.66c	17.00e	18.26e	18.43f
2-Sakha102	85.53d	85.00f	18.08c	17.66e	18.56e	18.46f
3-Giza178	90.23c	90.83e	24.00b	25.00c	20.03c	20.36d
4-Giza179	91.61c	91.83de	25.35b	25.33c	20.30c	20.45d
5-Sakha106	95.66b	95.41c	18.05c	18.33de	18.46e	18.11f
6-Sakha107	85.83d	86.08f	19.96c	19.33d	19.30d	19.33e
7-A22	98.33a	99.00a	28.00a	28.00b	21.06b	21.41c
8-E. Yasmine	98.25a	97.00b	19.00c	19.50d	24.40a	24.95a
9-EHR1	90.00c	92.83de	29.50a	29.83a	21.48b	21.98b
10-GZ9399	91.33c	91.66de	29.75a	30.16a	21.15b	21.30c
F. test	**	**	**	**	**	**
Interaction (V × T)	**	**	**	**	NS	NS

**Significant at 0.01 level. Means followed by a common letter at the same column are not significantly different according to Duncan^’^s multiple range at 0.05 levels.

Results revealed that theGZ9399and A22 produced the highest number of panicles/hills when compared at the same level of significance.. Meanwhile, the lowest values of the number of panicles/hills were recorded by Giza177 and Sakha102 in both seasons.

The interaction effects between planting method and rice genotype on plant height were significant in the 2020 and 2021seasons (Table 12).

**Table 12:** Plant height and number of panicle/hills affected by the interaction between the studied actors.

Treatment	Plant height (cm)				Number of panicle/hills	
	2020	2021	2020	2021	2020	2021
Planting methods (T)						
Rice genotypes (V)	T1	T2	T1	T2	T1	T2
1-Giza177	95.00c	76.00h	94.33d	77.33i	21.33ef	12.66h
2-Sakha102	95.40c	75.66h	95.00cd	75.00j	21.66ef	13.66h
3-Giza178	96.46c	84.00fg	97.00c	84.66g	28.66b	21.33ef
4-Giza179	96.56c	86.66ef	95.33cd	88.33f	30.33b	20.33f
5-Sakha106	101.00b	90.33de	99.83b	91.00e	23.33de	13.33gh
6-Sakha107	89.33e	82.33g	90.50e	81.66h	23.00def	15.66g
7-A22	105.66a	91.00de	104.33a	93.66d	30.33b	25.66cd
8-E. Yasmine	105.66a	90.83de	106.33a	87.66f	24.66de	14.33gh
9-EHR1	93.33cd	86.66ef	94.66cd	91.00e	36.00a	23.66de
10-GZ9399	94.66c	88.00e	95.3cd	88.33f	34.00a	26.33c
F. test	**		**		**	

T1 = Transplanting, T2 = Aerobic, **Significant at 0.01 level. Means followed by a common letter at the same column are not significantly different according to Duncan^’^s multiple range at 0.05 levels.

The tested genotypes exhibited great and marked differences under two planting methods. Rice varieties A22 and E. Yasmine showed the tallest plant under transplanting method, whereas under aerobic cultivation, only A22 showed the tallest plant. E. Yasmine showed the longest panicle under the transplanting and aerobic cultivation methods in both seasons. EHR1 and GZ9399 had high values for the number of panicles/hills under the transplanting method in both seasons. The lowest values of traits were obtained by Giza177 under aerobic conditions in both seasons.

Results indicated that the planting methods significantly affected the number of filled grain/panicle and the number of unfilled grains/panicles in both seasons (Table 13). The transplanting method produced the maximum number of filled grain/panicle, except for the number of unfilled grains concerning the highest value with aerobic cultivation in both seasons.

**Table 13:** Filled grains and unfilled grains/panicle numbers of some rice affected by the planting methods.

Treatment	No. of filled grain panical^−1^		No. of unfilled grain panical^−1^	
	2020	2021	2020	2021
Planting method (T)				
Transplanting rice	126.80a	127.41a	7.14b	8.23b
Aerobic cultivation	89.00b	88.71b	22.57a	24.00a
F. test	**	**	**	**
Rice genotype (V)				
1-Giza177	94.50f	93.16h	21.56a	22.08b
2-Sakha102	90.33g	90.76i	22.75a	24.66a
3-Giza178	125.33b	125.50c	8.00f	9.08f
4-Giza179	117.00c	118.66d	8.38f	9.00f
5-Sakha106	86.50h	86.66j	18.40b	20.66bc
6-Sakha107	97.16f	97.00g	14.66d	17.16d
7-A22	100.93e	102.50f	13.16e	13.66d
8-E. Yasmine	105.33e	105.66e	16.93c	17.33d
9-EHR1	134.00a	133.33a	18.33b	19.66c
10-GZ9399	128.00b	127.33b	6.33g	7.83f
F. test	**	**	**	**
Interaction (V × T)	**	**	**	**

**Significant at 0.01 level. Means followed by a common letter at the same column are not significantly different according to Duncan^’^s multiple range at 0.05 levels.

The rice genotypes significantly differed in their number of filled grain/panicle and number of unfilled grains in both seasons. EHR1 gave the highest values of the number of filled grain/panicle. However, the lowest value was recorded by Sakha102 and Giza177 in the two seasons. The number of unfilled grains reached the lowest values by GZ9399 cultivar. Meanwhile, the highest values of the number of unfilled grains were obtained with Giza177 and Sakha102 in both seasons. The interaction between planting methods and varieties significantly affected the number of filled grains and the number of unfilled grains in the two seasons (Table 14).

**Table 14:** Filled grains and unfilled grains/panicle numbers of some rice as affected by the interaction.

Treatment	No. of filled grain				No. of unfilled grain			
	2020		2021		2020		2021	
Rice genotype	T1	T2	T1	T2	T1	T2	T1	T2
1-Giza177	118.33e	70.66h	117.33f	69.00j	8.46ghi	34.66a	8.83ghi	35.33b
2-Sakha102	118.33e	62.33i	117.09f	64.44k	9.50e–h	36.00a	11.00fgh	38.33a
3-Giza178	141.00b	109.66f	142.00b	109.00g	4.00k	12.00e	4.83i	13.33f
4-Giza179	123.0de	111.00f	126.00d	111.33g	4.76jk	12.00e	4.66i	13.33f
5-Sakha106	117.66e	55.33j	118.33f	55.00l	7.13hij	29.66b	8.00hi	33.33b
6-Sakha107	118.33e	76.00g	119.33f	74.66i	5.66jk	23.66c	6.66i	27.66c
7-A22	121.7de	80.00g	123.00e	82.00h	6.66ij	19.66d	8.33hi	19.00e
8-E.Yasmine	131.33c	79.33g	131.00c	80.33h	10.20efg	23.66c	12.33fg	22.33d
9-EHR1	147.66a	120.3de	148.00a	118.66f	11.33ef	25.33c	12.66fg	26.66c
10-GZ9399	130.66c	125.33d	132.00c	122.66e	3.66k	9.00fghi	5.00i	10.66fgh
F. test	**		**		**			**

T1 = Transplanting, T2 = Aerobic, **Significant at 0.01 level. Means followed by a common letter at the same column are not significantly different according to Duncan^’^s multiple range at 0.05 levels.

The transplanting method gave the highest number of filled grains, which was obtained by EHR1 followed by Giza178. Meanwhile, the highest values of the number of unfilled grains were recorded by Giza177and Sakha102 followed by Sakha106 under aerobic cultivation (Table 14). Panicle weight, 1000-grain weight, and grain yield were greatly responded to planting methods (Table 15). Panicle weight, 1000-grain weight, and grain yield were significantly reduced as a result of low water supply under aerobic cultivation. The yield reduction under aerobic cultivation were 19.55 and 22.23% compared to transplanting method with sufficient water supply in the first and second seasons, respectively (Table 15). The heaviest panicle and 1000-grain, and the highest grain yield were produced by the transplanting method. Generally, the studied rice genotypes significantly differed in their panicle weight, 1000-grain weight, and grain yield in both seasons. The EHR1 rice variety gave the higher panicle weight and grain yield, followed by Giza179. At the same time, the lowest values were obtained by Giza177 and Sakha102 in both seasons. Meanwhile, the highest values of 1000 grain weight were produced by Sakha102 and Sakha106, and Sakha107 had the same statistical level.

**Table 15:** Panicle traits and grain yield of some rice affected by the planting methods and rice genotypes.

Treatment	Panicle weight (g)		1000-grain weight (g)		Grain yield (t ha^−1^)	
	2020	2021	2020	2021	2020	2021
Planting method (T)						
Transplanting rice (T1)	3.00a	3.01a	25.56a	25.40a	9.97a	10.09a
Aerobic cultivation (T2)	1.91b	1.97b	23.96b	24.15b	8.00b	7.82b
F. test	**	**	**	**	**	**
Rice genotype (V)						
1-Giza177	2.02ef	2.08ef	26.60b	26.86bc	7.90e	7.71f
2-Sakha102	2.05ef	2.02f	27.81a	27.58a	8.03e	8.10e
3-Giza178	2.56cd	2.60c	20.56f.	20.95g	9.76b	9.86b
4-Giza179	2.84b	3.15a	26.50b	26.61c	10.00ab	9.98b
5-Sakha106	2.00f	1.97f	26.68b	27.08abc	7.95e	7.86f
6-Sakha107	2.16e	2.18e	27.78a	27.35ab	8.78d	8.30d
7-A22	2.47d	2.45d	22.91d	22.60e	9.25c	9.18c
8-E. Yasmine	2.50cd	2.44d	21.26e	21.83f	8.46d	8.35d
9-EHR1	3.26a	3.25a	24.15c	24.03d	10.18a	10.33a
10-GZ9399	2.65c	2.71b	23.28d	22.86e	9.91ab	9.80b
F. test	**	**	**	**	**	**
Interaction (V × T)	**	**	**	**	**	**

**Significant at 0.01 level. Means followed by a common letter at the same column are not significantly different according to Duncan^’^s multiple range at 0.05 levels.

However, the lowest values of 1000 grain weight were obtained by Egyptian Yasmine in both seasons. The lowest values of grain yield were obtained by Giza177. GZ9399 and Giza178 occupied the second order after EHR1 and Giza179 considering rice grain yield, while A22, Egyptian Yasmine, and Sakha107 came in the third-order concerning rice grain yield in both seasons. As mentioned in Table 14, the interaction between planting methods and rice genotypes significantly affected panicle weight, 1000-grain weight, and grain yield in both seasons. The EHR1 and Giza179 variety gave the heaviest panicle weight with transplanting planting method. Meanwhile, the highest value of 1000 grain weight was found in Sakha107, 106, 102, and Giza177 varieties under the transplanting method. On contrary, the lowest values of panicle weight were obtained by Sakha106 with aerobic cultivation. Furthermore, the lowest values of 1000 grain weight were found in E. Yasmine variety with aerobic cultivation. The interaction between planting methods and rice genotypes significantly affected grain yield in both seasons (Table 16). The maximum values of grain yield were noted in EHR1and Giza179 varieties with the transplanting method. Nevertheless, the lowest values of grain yield were obtained by Giza177. Under aerobic pital letterrice cultivation, Giza179 produced the maximum grain yield without significant variation with these brought by GZ9399 and EHR1 as well as Giza178. The worst varieties under aerobic were Giza177, Sakah102 and Sakha106 as drought sensitive ones. Furthermore, A22, E. Yasmine and Sakha107 showed a medium level of grain yield under aerobic cultivation as a medium drought tolerant variety considering grain yield.

**Table 16:** Panicle traits and grain yield affected by the interaction between the study factors.

Treatment	Panicle weight g				1000-grain weight g			
	2020		2021		2020		2021	
Planting method (T)								
Rice genotype	T1	T2	T1	T2	T1	T2	T1	T2
1-Giza177	2.60cd	1.44ij	2.71ef	1.45j	27.90b	25.30d	27.90a	25.83cd
2-Sakha102	2.67c	1.43ij	2.62f	1.43j	28.76a	26.86c	28.33a	26.93b
3-Giza178	3.16b	1.96gh	3.20c	2.00h	20.43g	20.70g	20.63f	21.26f
4-Giza179	3.40b	2.29ef	3.83a	2.82def	26.63c	26.36c	26.73b	26.50bc
5-Sakha106	2.75c	1.25j	2.67f	1.27j	28.76a	24.60d	28.66a	25.50cd
6-Sakha107	2.70c	1.62i	2.72ef	1.65i	28.73a	26.83c	28.36a	26.33bc
7-A22	2.84c	2.10fg	2.88de	2.02h	23.50e	22.33f	22.73e	22.46e
8-E. Yasmine	3.16b	1.83h	3.02d	1.86h	22.40f	20.13g	22.63e	21.03f
9-EHR1	3.80a	2.73c	3.83a	2.67ef	25.10d	23.20e	24.96d	23.10e
10-GZ9399	2.89c	2.41de	2.98d	2.44g	23.33e	23.23e	23.16e	22.56e
F. test	**			**	**			**

Treatment	Grain yield							
	2020		2021					
Planting method (T)								
Rice genotype (V)	Transplanting (T1)	Aerobic (T2)	Transplanting (T1)	Aerobic (T2)				
1-Giza177	9.56c	6.23e	9.43cd	6.00i				
2-Sakha102	9.60c	6.46e	9.76c	6.43h				
3-Giza178	10.43b	9.10c	10.73b	9.00ef				
4-Giza179	10.63b	9.36c	10.76b	9.20de				
5-Sakha106	9.60c	6.30e	9.80c	5.93i				
6-Sakha107	9.46c	7.50d	9.50cd	7.10g				
7-A22	9.50c	8.60cd	9.66c	8.70f				
8-E. Yasmine	9.33c	7.60d	9.46cd	7.23g				
9-EHR1	11.07a	9.30c	11.23a	9.43cd				
10-GZ9399	10.53b	9.30c	10.50b	9.10de				
F. test	**		**					

T1 = Transplanting, T2 = Aerobic, **Significant at 0.01 level. Means followed by a common letter at the same column are not significantly different according to Duncan^’^s multiple range at 0.05 levels.

## Discussion

Aerobic rice cultivation is a medium option to save water with an insignificant yield reduction for irrigation rice ecotypes^6^.

### Antioxidant enzymatic activity

From going findings, the tested rice genotypes were at par regarding the antioxidants plant content under the transplanting method (well watering) because gene expression less and low free radical formation. Under aerobic conditions, there are great variations among genotypes with respect to their ability to produce more antioxidant against free radicals development under such stress. Rice genotypes of EHR1, Giza179, GZ9399, Giza178 and A22 had high concentration of enzymatic antioxidants comparing others under the same conditions and particularly under aerobic condition in the terms of low water input. On contrary, Giza177, Sakha102 and Sakha106 showed weakness to release reasonable concentrations of antioxidants under aerobic conditions (stress case) even comparing to their concentration under transplanting. Oxidative damage by releasing reactive oxygen species (ROS) under abiotic stresses; high and low temperature, salt stress, water stress, and water dehydration represents a negative effect on plant growth and yield. One of the unavoidable consequences of drought stress is raising ROS production in the different cellular compartments, namely in the chloroplasts, the peroxisomes and the mitochondria. Drought caused greater inhibition of photosystem II quantum efficiency, carboxylation efficiency, and photosynthetic capacity parameters as a result of more accumulation of reactive oxygen species (ROS)^37,38^. The attach of ROS attack and react with the cell membrane's phospholipids, reducing its stability and causing a high EL% of the cell^39^. ROS in plants was removed by a variety of antioxidant enzymes and/or lipid-soluble and water-soluble scavenging molecules, the antioxidant enzymes being the most efficient mechanisms against oxidative stress^40–45^. Drought stress greatly influences physiological and biochemical functions that affect plant growth^46–49^. Catalase (CAT), peroxidase (POX) and superoxide dismutase (SOD) increase in under drought conditions. These antioxidant enzymes can protect cells from oxidative damage. Similarly, proline synthesis increased under drought conditions, showing that proline could also act as part of a survival mechanism under drought^50^. Moreover, drought leads to an increase in proline accumulation, which proline helps to maintain tissue water status. Accordingly, rice plants adapted to drier environments have increased the activity of antioxidant enzymes to eliminate ROS^13^. Continuously, the varieties; EHR1, Giza179, GZ9399, Giza178, and A22 showed apparent capacity to exert an avoidant concentration of proline under aerobic cultivation (stress conditions), which acts as an osomo-protectant and water supplier under drought. The other genotypes including Giza177, Sakha102 and Sakha106 had low efficiency to accumulate sufficient level of proline to enable them to grow healthy under aerobic conditions, indicating their drought sensitivity. Like antioxidants, all tested rice entries were at the same level of significance regarding to the proline accumulation under the transplanting method (well watering). Therefore, the ability of rice genotype to accumulate continuously reasonable concentrations of both proline and antioxidants could be recognized as valid genotype under aerobic conditions.

### Physio-morphological traits

Water deficit causes negative effects on the enzymes activities resulting in many changes in plant tissues, cell damage, nutrient uptake and photosynthesis rate^51^. Aerobic cultivation significantly decreased the measured physio-morphological such as leaf area index, flag leaf area, and chlorophyll content, but increased leaf rolling. The ten rice genotypes differed regarding the change rate under aerobic conditions owing to their adaptation capabilities. Like the antioxidant concept, Giza179, EHR1, GZ9399, Giza178 and A22 as one resilience category, showed higher leaf area index, flag leaf area, chlorophyll content and low leaf rolling combined with high stomata conductance under aerobic conditions irrespective of transplanting method. The high physio-morphological concept possessed by the resilience group might be reflected on raising its adaption, growth, photosynthesis, development and finally sink. On contrast, the worse category varieties including Giza177, Sakha102 and Sakha107 brought severe reduction in its physio-morphological traits along with high leaf rolling indicating its invalidation under aerobic conditions. It is important to address that high leaf roiling is a sensitivity indicator, not tolerance, which was matched with poor growth. Furthermore, the reduction in chlorophyll of sensitive varieties might affect the growth and physiological events such as photosynthesis and energy synthesis. Generally, the tolerant group keeps reasonable root length with proper volume under aerobic conditions comparing to other group which failed to give a good root system. It became clear that good root system is an essential option for validation under aerobic conditions. High leaf rolling for sensitive group might be affected the stomatal conductance and subsequently reducing gas exchange and restricting photosynthesis. One of the essential defense mechanisms in plants is avoiding drought, by adjusting some plant attributes, such as the reduction of leaf rolling or leaf size.

### Cell membrane stability

It is well known that the conservation of integrity and stability of membranes under water stress is a major component of drought tolerance in plants^52^. Cell membrane stability diminished rapidly in Kentucky bluegrass exposed to drought and heat stress simultaneously^53^. The decrease in chlorophyll and increase in EL% may be due to the role of free radicals in damaging the membrane phospholipids of the cell wall and plant pigments. The antioxidant enzymes are the most efficient mechanisms against oxidative stress. Apart from catalase, various peroxidases and peroxiredoxins, four enzymes are involved in the ascorbate–glutathione cycle, a pathway that allows the scavenging of superoxide radicals and H_2_O_2_^27^. The cell membrane stability was reduced under aerobic conditions, but the genotypes that possessed high concentrations of measured antioxidants under the current study kept high capability of cell membrane without damage such as GZ9399, Giza179 and EHR1. The previous varieties had low stability index, since a low index is favorable that indicating a low EL%. The low EL% provided the ability of these varieties to create satisfied defense system like antioxidants. The antioxidant could eliminate the hazard free radical developed under drought stress, which damaged the cell membrane^54,55^. The results pertaining to sensitive varieties such as Giza177, Sakha102 and Sakha106 confirmed the previous concept, which had a high stability index that matched the low concentration of antioxidants indicated apparent cell membrane damage. The damage of cell membrane induced more EL% of plant cell that was hold true with drought sensitive varieties such Giza177. Proline is acting as antioxidant and as osmo-protectant material under abiotic stresses, particularly water deficit and salt stresses^11^. Improved water status may be achieved through osmotic adjustment and/or changes in cell wall elasticity that results in maintaining physiological activity for extended periods of drought. Giza179, GZ9399 and EHR1 showed high proline accumulation that was completely matched with a high RWC under aerobic conditions.

### Stomatal conductivity

The fast and first response of plants to water deficit is the closure of their stomata to minimize the transpiration of water. Recently, stomatal closure was generally accepted to be the main determinant for decreased photosynthesis under mild to moderate drought^56^. Again, EHR1, GZ9399 and Giza179 might have a high affinity to regulate stomata conductance under aerobic conditions with high efficiency that might be due to their ability to do balance between the K and ABA concentration in the guard cells. Furthermore, The mentioned genotypes as resilience one based on previous findings showed less leaf rolling under aerobic conditions, reflecting on optimum regulating of stomata conductance and subsequently CO_2_ sufficient supply needed for photosynthesis. The group including Giza177, Sakha102 and Sakha106 exhibited low stomata conductance owing to sharp leaf rolling for a long time under aerobic conditions and poor adaptation. Meanwhile, aerobic cultivation showed a reduction in such traits with various levels according to the adaptation abilities of varying genotypes. Less water input might have increased water loss through stomata, and increased respiration rates resulted in a reduction of the most of the physiological processes and photosynthetic rate^57^. Rice plants adapted to dry environments develop fewer stomata on the surface to avoid losing more water and growth limiting, also, decreased transpiration rate as a function enhanced the drought tolerance. The management of stomata conductance is one of the most important parameters for plant resistance under stress.

### Yield and yield components

Aerobic conditions with low water input might reduce cell division, elongation, and plant height as well as panicle length; also, low water input resulted in a low number of panicle/hill^32^. The previous concept was found under aerobic conditions comparing to transplanting method (well water supply). This may be attributed to the role of water and nutrients for producing new tillers and total number of grains/panicle^6^. Aerobic cultivation significantly reduced the number of filled grains combined with increasing unfilled grain, but the tolerant varieties; Giza79, EHR1, GZ9399, A22, and Giza178 were less affected comparing to another opposite group involving Giza177, Sakha102 and Sakha106. Low water supply under aerobic conditions might affect metabolism, development, photosynthesis assimilates and filled grains. Giza179, EHR1, GZ9399, A22 and Giza178 were found effective to developed different drought mechanisms, which reflected on their healthy growth and contentment sources that ensure proper sink along with heavy grain (panicle). Well sources and sink of tolerant rice genotype brought acceptable grain yield under aerobic conditions. The opposites was corrected with well watering under transplanting regarding plant development, physiological processes such as photosynthesis, enzyme activity, which assimilates transportation from source to sink, resulting in improved yield and yield components of rice^58^. Grain yield was reduced as a result of lower amounts of water supply. The reduction in grain yield might be attributed to declining in 1000 grain weight and spikelets/panicle^51^. Water deficit during early flowering reduced the number of fertile spikelets and resulted in decreased final grain yield production, this might also be associated with the reduction in leaf area, lower photosynthetic rates, and assimilate transportation from source to sink and dry matter partitioning^59^.

## Conclusions

It is concluded that water deficit stress greatly influenced physiological functions and biochemical activities that negatively affect plant growth. Superoxide dismutase and catalase as well as, proline and RWC increased under drought stress condition, particularly with tolerant varieties, indicating that the tolerance to aerobic stress is not imputable to a single component, but it is a multifactorial response. Thereby, based on the performance of growth, biochemical, yield, and yield attributes, Giza179, GZ9399, EHR1 and A22, and Giza178 is recommended to lead cultivation under aerobic conditions.

## Supplementary Information

Supplementary Figures.

## Data Availability

Data used in the current research presented in the manuscript. The source of seeds is Rice Research and Training Center, Giza, Egypt.
