# Supplementary material for: Response of varied rice genotypes on cell membrane stability, defense system, physio-morphological traits and yield under transplanting and aerobic cultivation
Source: Sci Rep. 2023 Apr 8;13:5765. doi: 10.1038/s41598-023-32191-6 (PMC10082820; doi:10.1038/s41598-023-32191-6)
Supplement: Supplementary file 1 — Supplementary Figures. [file 41598_2023_32191_MOESM1_ESM.docx]

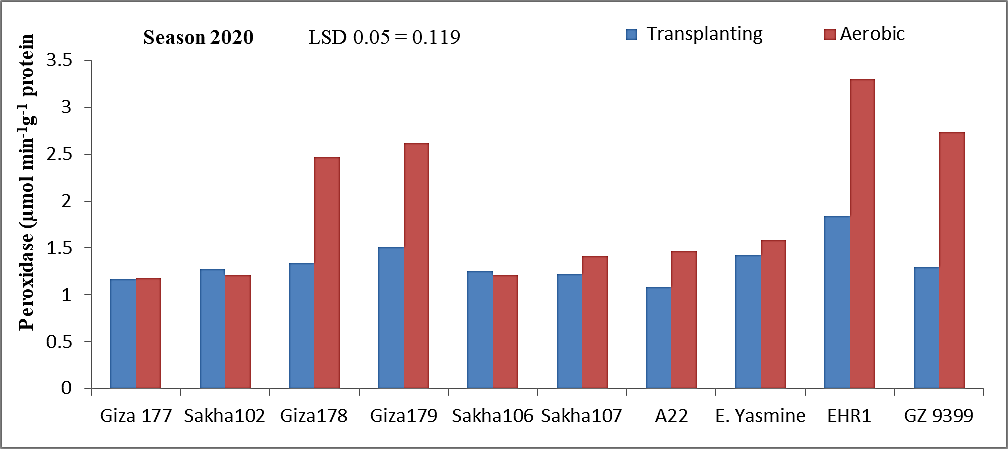


Figure S1. Peroxidaseas affected by the interaction between genotypes and planting methods in 2020 season.


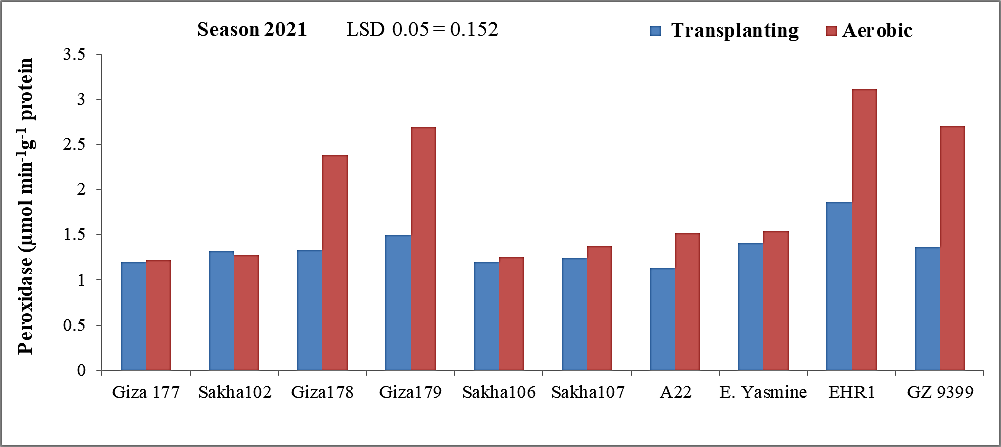


Figure S2. Peroxidase as affected by the interaction between genotypes and planting methods in 2021 season.


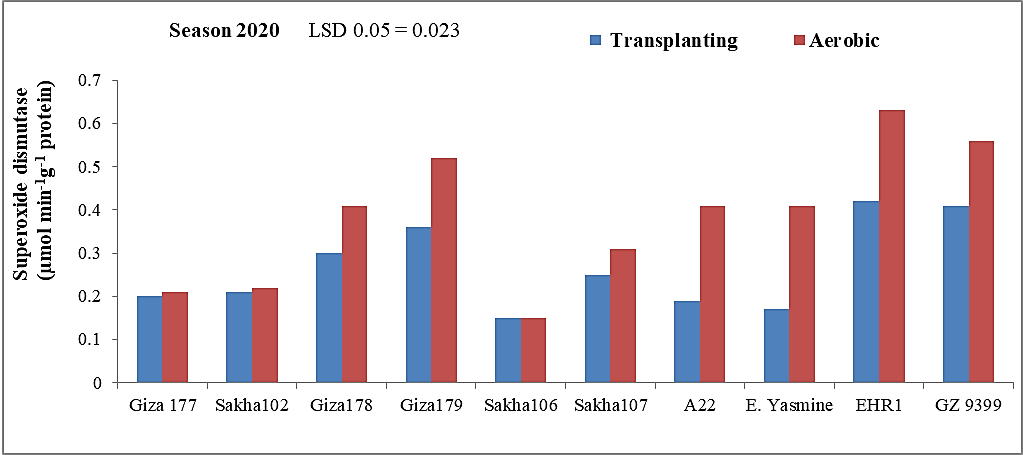


Figure S3. Superoxide dismutase (µmol min^-1^g-1 protein) as affected by the interaction between genotypes and planting methods in 2020 season.


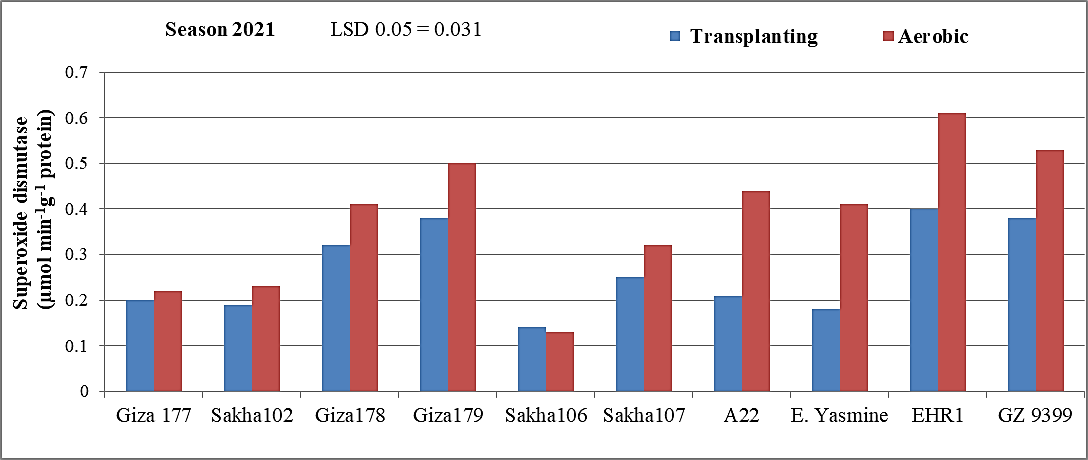


Figure S4. Superoxide dismutase (µmol min^-1^g^-1^ protein) as affected by the interaction between genotypes and planting methods in 2021 season.


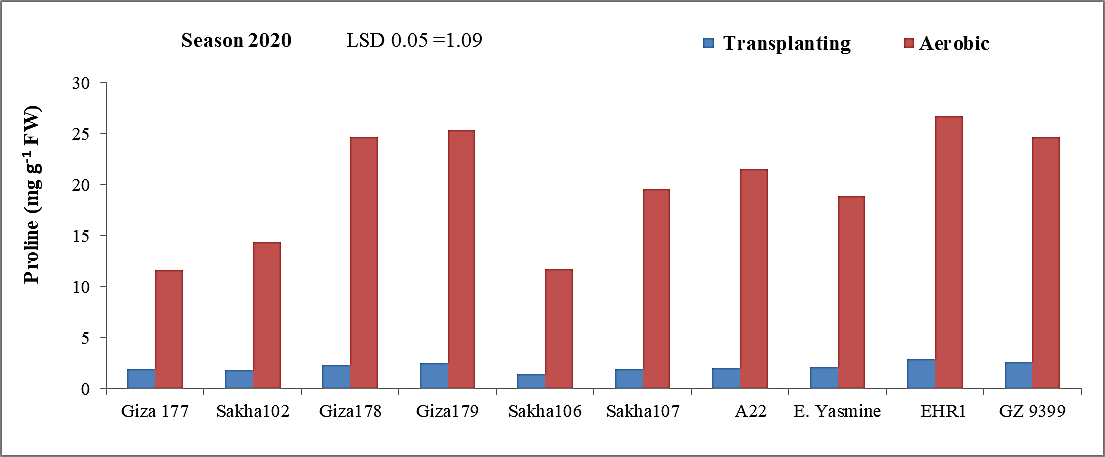


Figure S5. Proline (mg g^-1^ FW) as affected by the interaction between genotypes and planting methods in 2020 season.


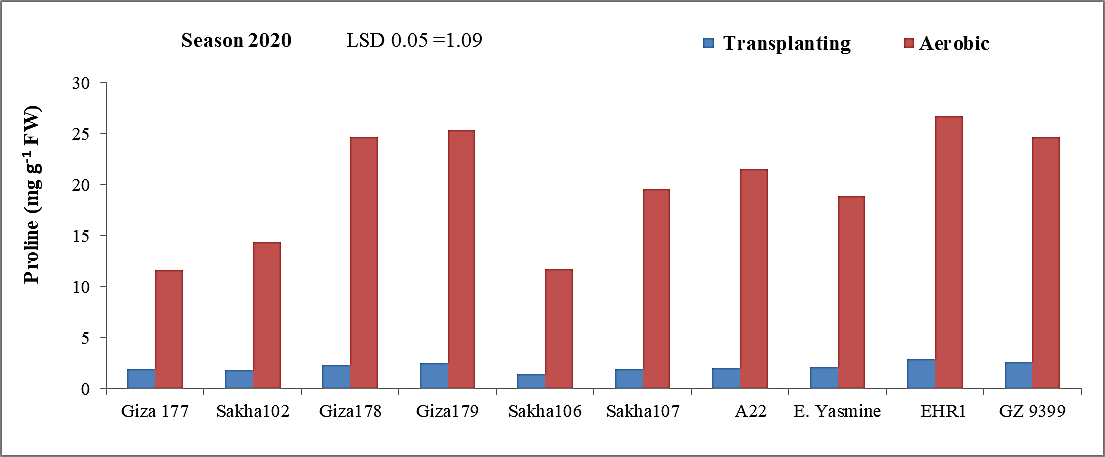


Figure S6. Proline (mg g^-1^ FW) as affected by the interaction between genotypes and planting methods in 2021 season.


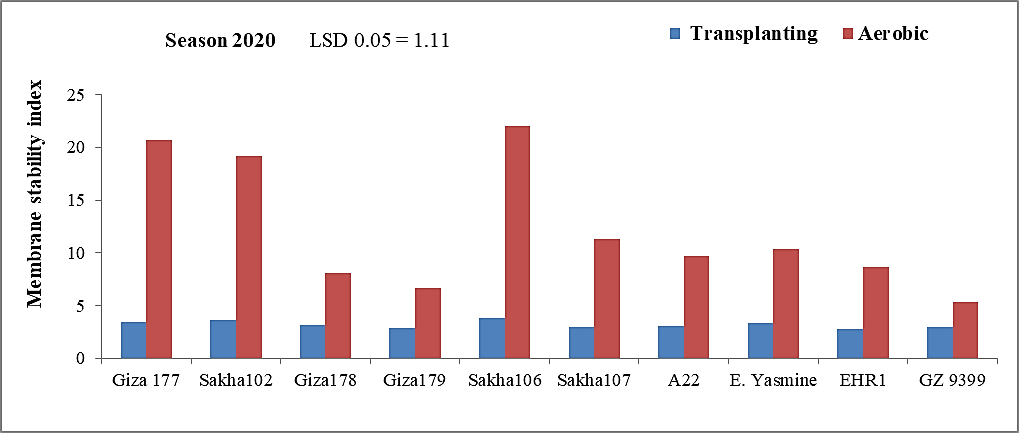


Figure S7. Membrane stability index unit as affected by the interaction between planting methods and rice genotypes in 2020 season.

**
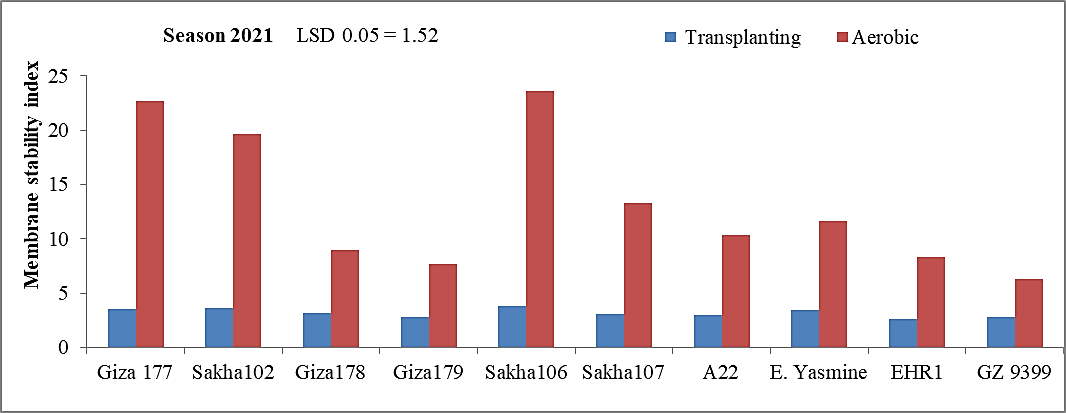
**

Figure S8. Membrane stability index unit as affected by the interaction between planting methods and rice genotypes in 2021 season.
